# Biting Flies and Associated Pathogens in Camels in Amibara District of Afar Region, Ethiopia

**DOI:** 10.1155/2024/5407898

**Published:** 2024-01-09

**Authors:** Adugna Chalchisa, Bersissa Kumsa, Fekadu Gutema Wegi

**Affiliations:** ^1^College of Veterinary Medicine, Haramaya University, P.O. Box 138, Dire Dawa, Ethiopia; ^2^College of Veterinary Medicine and Agriculture, AAU, Addis Ababa University, P.O. Box 1174, Addis Ababa, Ethiopia; ^3^Ethiopian Institute of Agricultural Research, Holeta Agricultural Research Center, Holeta, Ethiopia

## Abstract

Biting flies and associated pathogens are the major health constraints on camel production and productivity and are implicated in causing significant economic losses in the pastoralist community in Ethiopia. A cross-sectional study was conducted to estimate the prevalence of biting flies and their associated pathogens in relation to different risk factors in camels in the Amibara district, from October 2019 to April 2020. A total of 480 camels were examined for biting flies and associated pathogens. The study revealed that overall, 87% (418/480) and 18% (87/480) of camels were infested by one or more biting flies and infected with *Trypanosoma evansi* during the study period, respectively. The collected biting flies were identified into a total of 3 genera: *Hippobosca, Stomoxys*, and *Tabanus* under the stereomicroscope. In the present study, *Hippobosca* (40.4%) was the most prevalent biting fly, followed by *Stomoxys* (31%) and *Tabanus* (28.6%), which affected camels in the study area. Among camels infected with *Trypanosoma evansi*, 7.3% and 16% were positive for parasitological and serological tests, respectively. Age, body condition score, and season appeared to have a significant effect (*p* ≤ 0.005) on the prevalence of biting flies and *T. evansi* on dromedaries. According to the findings of this study, biting flies and *Trypanosoma evansi* were the most common limitations on camel health, production, and productivity in the study area. As a result of the possible threat of biting flies' infestation and *Trypanosoma evansi* on camels, all-around attention is required in terms of strategic acaricide application, proper antiprotozoal drug use, and raising knowledge about acaricide use to prevent and control biting flies' infestation.

## 1. Introduction

Ethiopia has the largest livestock population in Africa, but its contribution to the country's economy is still low, and diseases are a major constraint [1]. Livestock population in Ethiopia is estimated at 70 million cattle, 57 million poultry, 52.5 million goats, 42.9 million sheep, 10.8 million donkeys, 8.1 million camels, 2.15 million horses, and 0.4 million mules [2]. In addition to producing milk, meat, and skins and hides, animal resources play an important role in providing export commodities, such as live animals, hides and skins, and honey, contributing 8.3 percent of total GDP and about 20.2 percent of agricultural GDP [1].

According to the Central Statistical Agency's [3] report, Ethiopia is ranked sixth in Africa in terms of its camel population. The distribution of camels is significantly influenced by their ability to withstand extreme heat and desiccation. Camels are normally distributed throughout subtropical dry areas in Africa and Asian countries [4]. In Ethiopia, camels are found in arid and semiarid parts of the southern, eastern, and northeastern parts of the country, mainly in the Borana, Ogaden, and Afar regions [5].

Dromedaries, the one-humped camel (*Camelus dromedarius*), is the only type of camel that exists in Ethiopia [6]. *Camellus dromedaries* are an important animal's species in the pastoral economy in Ethiopia because of their extraordinary ability to perform in arid and semiarid environments where there is scanty vegetation, which is not sufficient for other livestock species. The camel is a multipurpose animal especially adapted to arid and semiarid environments, enabling nomadic peoples of the world to live in difficult environments. Camel is primarily kept for milk production, meat production, draft power, transportation, and as a draft animal in agriculture [7]. It also serves as a financial reserve and has a significant impact on social prestige and wealth. Despite its significant contribution to the pastoralist society's livelihood, there is very little scientific information on the health and productivity of camels in Ethiopia [8].

A slow reproduction cycle, high calf mortality, and other health problems are among the major constraints contributing to the decreasing camel herd population and productivity [9]. Ectoparasites are accountable for decreased camel production and reproduction, as well as leather quality deterioration, downgrading, and skin rejection [10]. Biting flies and their associated pathogens are important constraints to the production, productivity, and performance of camels and other animals [11].

*Tabanus* and *Stomoxy* are hematophagous insects most commonly reported from camels and are responsible for noncyclical transmission of *trypanosomes* in camels in various parts of the world. *Trypanosoma evansi* is mechanically transmitted in camels by the bites of haematophagous flies such as *Tabanus and Stomoxys* [12]. The essential biting flies for the spread of *T. evansi* are *Tabanus* species [11]. Biting flies belonging to the genera, *Tabanus*, *Stomoxys*, *Chrysops*, *Hippobosca*, and *Lyperosi*a, have been reported to affect camels in Ethiopia [13, 14]. Rainfall, moisture-retaining clay soil, and surface water pools where *Acacia senegal* shrubs grow abundantly are all conducive to the vector's survival and reproduction [11].

One of the most common diseases in camels is trypanosomosis [13, 15]. This disease is caused by *Trypanosoma evansi*, which is mechanically transmitted by hematophagous biting flies, particularly *tabanids* [13]. The disease causes a significant productivity loss in camel herds and even mortality in the acute form [15]. Trypanosomosis is widespread throughout the world and a major constraint to camel productivity [13, 16, 17].

Early detection and taking major action are important to control blood-sucking arthropods instead of waiting till the problem gets severe [18]. Biting flies can be effectively controlled by avoiding the flies' habitat, avoiding heavily wooded and grassy areas, using repellents, and performing frequent fly checks before they can transmit disease [19]. Regular studies on the dynamics and species composition of biting flies and other parasitic arthropod populations, coupled with the present efficacy status of acaricides against the most prevalent and economically pertinent biting flies and other parasitic arthropod species of an area, are necessary to carry out efficient control [20]. A comprehensive understanding of the genus identity, composition, seasonal dynamics and variation, and epidemiology is critical for preventing and controlling economically important parasitic arthropods [18].

Despite the existence of a large population of camels in Ethiopia and their great social and economic importance to the pastoralist community, there is no documented information on the genus of biting flies in camels, their associated pathogens, and associated risk factors for camels in the study area. As a result, the current study was designed with the objective of estimating the prevalence of biting flies and their associated pathogens in relation to different risk factors in camels in the Amibara district.

## 2. Materials and Methods

### 2.1. Description of the Study Area

The Afar National Regional State is found in the eastern region of the country, situated between 39° 34′ to 42° 28′ E longitude and 8° 49′ to 14° 30′ N latitude. The region covers approximately 270,000 square kilometers in terms of its overall geographic size, and over 90% of the people living there are engaged in pastoralism. The other 10% consists of agropastoralists and commercial farmers who have recently emerged due to the development of small-scale irrigation along permanent and temporary rivers. The area is situated alongside Eritrea in the northeast and Djibouti in the east. It is also delimited by the regional borders, with Tigray Regional State to the northwest, Amhara to the southwest, Oromia Regional State to the south, and on the southeast with the Somali Region of Ethiopia [21].

The climate of Afar Regional State is arid and semiarid, with low and inconsistent rainfall. The region's altitude ranges from 120 meters below sea level to 1500 meters above sea level. The temperature ranges from 20°C at higher elevations to 48°C at lower elevations. The region's rainfall is bimodal, with a mean annual rainfall of less than 500 mm in the semiarid western escarpments and less than 150 mm in the arid zones to the east. According to the Central Statistical Agency of Ethiopia (CSA), the region has a total population of 1.8 million people and is administratively divided into five zones, 34 administrative districts, and 358 pastoral associations (PA) [21].

Amibara district is located about 250 km to the northeast of Addis Ababa and has 18 PAs (the least administrative units) with total population of ∼63,378, of which 35,374 are men and 28,004 women. The districts have 2 small urban and 30 rural PAS. The livestock populations of the Amibara district are composed of 103, 959 cattle, 122, 526 goats, 48,043 sheep, 3,888 donkeys, and 39,995 camels [21].

**Figure 1:**
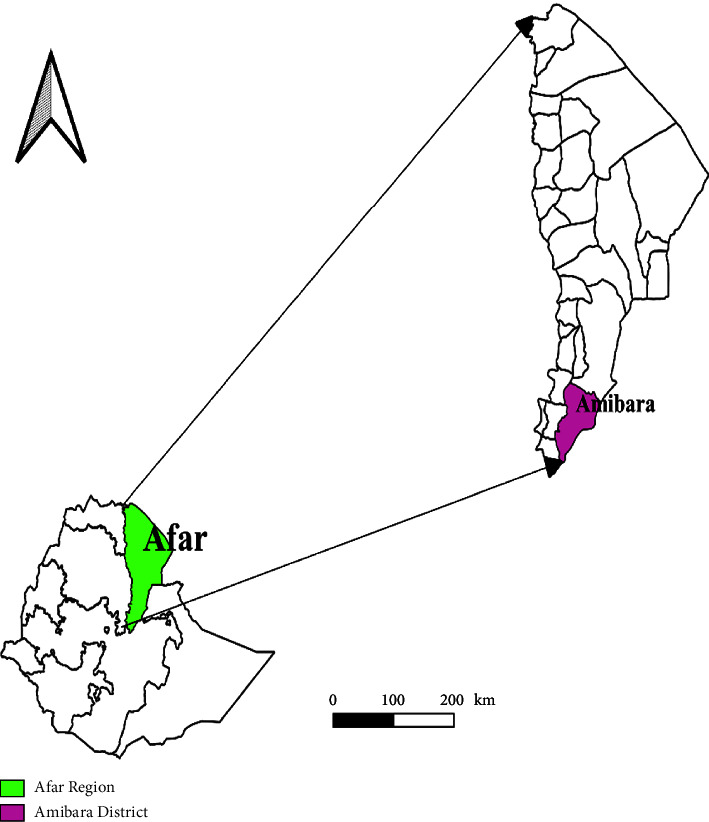
Map of Ethiopia showing the present study area. Source: Diva GIS.

The current study was conducted in the Amibara district (Figure 1) of the Afar region's Gabi Rasu zone (zone 3) in the Middle Awash Valley. This study area was selected purposefully based on the number of camels found in the area. The district has arid and semiarid agroecologies, and livestock production is the community's main occupation. The distribution of rainfall is inconsistent and occurs in two distinct periods. The first period, known as the long rainy season (kerma), happens from mid-June to mid-September. The second period, called the short rainy season (Sugum), takes place between March and April. The typical yearly precipitation ranges from 400 to 600 mm.

### 2.2. Study Population

The study animal consists of camels of all ages and sex groups residing in the Amibara district of the Afar region. Individual camels selected with different criteria were included in the study. The following information was collected: age, sex, body condition, season, herd composition, herd size, and previous treatment history. In the study area, certain camels are coherded (kept) with cattle, sheep, and goats. Body condition was determined using the references by the authors in [22, 23], who classified dromedary body condition scores as poor, medium, or good.

This study included a total of 480 camels of various ages and both sexes. All camels included in this study were examined for biting flies and associated pathogens. Samples were collected from 160 animals during each of these three seasons: the cooler weather/wet/Gilal (November-December), shower/dry (Daba) January-February, and short rain/Sugum (March-April) seasons for seasonal variation of biting flies' distribution and their associated pathogens [24].

### 2.3. Study Design and Sampling Techniques

From October 2019 to April 2020, a cross-sectional study was conducted with the primary objective of identifying the genus of biting flies infesting camels and their associated pathogens, estimating their prevalence, and assessing risk factors associated with biting flies' infestation and associated pathogens in the study district. The current study included only camels that had not been treated with any acaricides and antiprotozoals, for a month prior to sample collection.

According to Toma et al. [25], a combination of convenience and purposive sampling methods was used. First, the study district was chosen based on its camel population and accessibility to the area. A complete list of pastoral villages (sampling frame) was obtained from eight Amibara pastoral associations (PAs) chosen based on accessibility. Three to six herds have been selected from among the volunteer herds. The sampling method for camel herds was also purposeful (based on the owners' willingness) and involved simple random selection for the individual study animals. Depending on the number of camels available in each herd, an average of 8 (7–9) from herd sizes less than 20 (twenty), 15 (10–20) from herd sizes 20–40 (twenty to forty), and 25 (20–30) from herd sizes greater than 40 (forty) were randomly selected from each herd. Each of the animals selected as sampling units was examined for biting fly infestation and was categorized as positive or negative based on the presence or absence of biting flies on the animals' bodies.

### 2.4. Sample Size Determination of the Study Camels

Because no previous estimate of the prevalence of biting flies' infestation and associated pathogens in the district existed, the sample size was calculated using a 50% expected prevalence, a 5% desired absolute precision, and a 95% confidence level or interval (CI) [26]. Using the previous formula provided by [26] to calculate sample size (*n*) is as follows:

(1) $$\begin{array}{c} n=\frac{1.96^{2}\;x\text{ Pex }x\;\left(1-\text{Pex}\right)}{d^{2}}, \end{array}$$

where *n* = sample size, *d* = desired absolute precision (0.05), and Pex = expected prevalence (50%); thus, the desired sample size for Pex = 0.5 is *n* = 384. As a result, 384 were considered the minimum sample size required for biting fly infestation and their associated pathogens at the study site. However, to increase precision, a total of 480 camels from the district were examined for the presence of biting flies and for other associated pathogens during the whole study period. Accordingly, 160 camels were selected purposively from the district in each dry, wet, and short rainy season for biting flies as well as for the detection of associated pathogens.

### 2.5. Sample Collection and Laboratory Processing of Biting Flies

In addition to the parasitological studies, biting flies were also collected from camels and the surrounding area to see if any of them could be used as vectors for the spread of *Trypanosoma evansi* and *Trypanosoma Vivax*. As it was believed to be representative of other comparable study localities, this collection was made in order to observe changes in the fly population over the course of various seasons. Flies that were actively feeding on the animals and hovering in the area of the animals were captured in the study area. Aerial sweeping nets were used to collect the flies from camels and their surroundings while they were flying (circling the area around the animals), as described by Barros and Barros [27]. To put it briefly, the person holding the net walks beside the animals and sweeps it periodically to capture flies that are feeding on the animals and flying around the animals. Aerial sweeping nets were used to catch biting flies from camels while they were being fed, especially during the morning when the flies were actively feeding, resting, and circling on the camels. After each sampling, the nets used for collection were emptied before the next collection. The total number of flies collected was counted and then transferred into a sample bottle. On every specimen that was collected in the field, the following information was noted: location, date, season, and time of collection. The captured flies were kept in sample bottles (screw-capped bottles) that were filled with 70% ethanol. The sample was transported to the Veterinary Parasitology Laboratory at the College of Veterinary Medicine and Agriculture, Addis Ababa University, using these screw-capped bottles. In the laboratory, the process of identifying flies involved placing the sample on a Petri dish by using forceps. Through the use of standard identification keys, the adult flies were then identified to the level of genus under a stereomicroscope and counted manually under a stereomicroscope. Identification was done based on taxonomic features such as the arrangement of their wings, mouth parts, antennae, size of flies, the patterns on their abdomen, their color, and the separation between their eyes as described by [28–30]. References [31, 32] were used to identify the genus Stomoxys. The genus *Tabanus* was identified using references [33, 34]. References [35, 36] were used to identify the genus *Hippobosca*.

### 2.6. Blood Sample Collection and Examination

Whole blood samples from 480 camels were collected via jugular vein puncture into 10 ml ethylene tetra-acetic acid (EDTA) and plain vacutainer tubes. The Buff coat technique has been employed immediately after blood collection to determine the level of parasitemia and anemia at the collection site. The blood was immediately transferred into capillary tubes, sealed at one end, and centrifuged at 12,000 rpm for 5 minutes using a microhaematocrit centrifuge [37]. After that, each capillary tube was placed in a haematocrit reader, and the reading was expressed as a percentage of packed red cells to total volume (PCV) of whole blood. The level of anemia was determined using a cutoff PCV value of 27 and classifying animals with a PCV value less than 27 as anaemic and those with a PCV value greater than 27 as nonanaemic [37].

Buff coat method: with a diamond-tipped pencil, the capillary tube was cut 1 mm below the buffy coat. The contents of the capillary tube were poured onto a clean slide and thoroughly mixed. It was covered by a 22 × 22 mm cover slip. A bright-field microscope was used to examine the preparation, with the condenser top out and the diaphragm closed. For 30 minutes, the fixed blood smears were immersed in Giemsa stain solution in an upright position. After removing the stain, the slide was thoroughly washed in running tap water and allowed to drip-dry in an upright position before microscopic examination. At 100x magnification, the slides were examined under a microscope with oil immersion. According to Murray et al. [28, 37], species identification was based on *trypanosome* morphological characteristics such as trypanosome size, posterior end shape, kinetoplast size and position, and flagellum presence or absence.

Thin blood smears were prepared using the method described by Basaznew et al. [38]. Smears were air-dried and fixed in absolute methyl alcohol for 2-3 minutes. The slides were stained with Giemsa for 20–25 minutes before being washed with tap water to remove excess stain. After air-drying, the slides were examined using an oil immersion objective lens (100x) to detect and identify *Trypanosoma* species [38].

#### 2.6.1. Card Agglutination for Trypanosomosis Test (CATT/*T. evansi*)

The card agglutination for trypanosomosis test (CATT/*T. evansi*) was used to investigate the seroprevalence of camel trypanosomes. The CATT/*T. evansi* test is a direct, rapid card agglutination test that employs formaldehyde-fixed, freeze-dried *trypanosomes* stained with Coomassie blue and expressing a predominant variable antigen type of *T. evansi* (RoTat 1.2). Positive samples were determined at cutoff point dilutions of 1 : 4 and higher [39, 40]. As a result, 25 *μ*l of camel serum was pipetted onto the reaction zone of a plastic-coated test card after being diluted 1 : 4 in CATT buffer. After adding one drop (about 45 *μ*l) of CATT reagent, the reaction mixture was spread out with a stirring rod and allowed to react for 5 minutes at 70 rpm on a card test rotator. Blue granular deposits show a visible positive reaction to the naked eye [41].

### 2.7. Data Analysis

The collected data were entered and saved in the database using the Microsoft Excel spreadsheet (MS-2007) program. All the data were summarized, compiled, and coded before being stored in a Microsoft Excel 2007 spreadsheet. For statistical analysis, the data from MS Excel were imported into STATA version 13.0 statistical software (Stata Crop, 2013) and R software, Version 3.5.1. R Software, Version 3.5.1, was used to compute descriptive and analytic statistics. Descriptive statistics were used to summarize the data, which was then displayed in tables. Poisson regression was employed to analyze the number (count) of biting flies on animals as a function of various explanatory variables (sex, age, body condition score, herd size, herd composition, and season).

Furthermore, logistic regression was used to investigate the degree of association between a binary outcome (the presence or absence of biting flies) and various explanatory factors. The chi-square tests were used to quantify the relationship between the variables and the presence of a biting fly infestation. To determine prevalence (percentage), a statistical analysis was performed. Kappa statistics (K) were used to determine the level of agreement between diagnostic tests. The chi-square test, on the other hand, was used to examine the relationship between trypanosomosis positive camels in both tests and the assumed risk factors. Furthermore, the two-sample *t*-test was used to compare the mean PCV of parasitologically positive and negative camels, as well as serologically positive and negative camels, for trypanosomosis. For all statistical analysis, a statistical significance level of *p* ≤ 0.005 was considered.

### 2.8. Ethical Consideration

Ethical approval was obtained from the Animal Research Ethics and Review Committee (certificate Ref. No: VM/ERC/23/01/12/2020 from the College of Veterinary Medicine and Agriculture, Addis Ababa University, Bishoftu, Ethiopia) for the collection of blood samples from camels in an ethical manner. Camel sample collection did not jeopardize the animals' welfare. The camel owners were informed about the study's purpose, risks, and benefits based on their level of understanding in order to provide complete information, including the duration of the study, biting flies, and blood sample collection from the camels.

## 3. Results

### 3.1. Biting Flies' Infestation

Of the total of 480 camels examined for fly infestation, 418 (87%) of them were infested with one or more biting flies. Overall, three genera of biting flies, Hippobosca, Stomoxys, and Tabanus, were encountered during the study period. Morphological identification of the collected biting flies revealed that *Hippobosca* (40.4%) was the most prevalent and predominant genus in the Amibara district, followed by *Stomoxys* (31%) and *Tabanus* (28.6%) as shown in Table 1.

Assessment of the abundances of the collected biting flies showed that a total of 1,706 adult flies (1,051 female flies and 655 male flies) were collected during the study period. The number of biting flies that were collected from camels in the current study area during the wet season (536), short rain season (723), and dry season (447) is shown in Table 2. Moreover, the current study's findings revealed that biting flies were present on camels throughout the study period. All biting fly genera were abundant during the short rainy season and scarce during the dry season (Table 2).

The prevalence of biting flies on camels of different ages, sex, body condition score, herd composition, season, and herd size is shown in Table 3. Among the risk factors considered, age and season appeared to have a significant effect (*p* < 0.05) on the prevalence of biting flies on camels. However, the prevalence of fly infestation did not significantly differ among camels of different sex, body condition score, herd composition, and herd size (*p* > 0.05) (Table 3).

### 3.2. *Trypanosoma evansi* in Camels

The card agglutination test indicated an overall seroprevalence of 16% (77/480) for the detection of agglutinating antibodies against *Trypanosoma evansi* (Table 4). Parasitemia ranged from a few parasites per slide to over five trypanosomes per field on the microscope field. The overall parasitological prevalence of *Trypanosoma evansi* in the Amibara district of the Afar region was 7.3% (35/480) (Table 4).

The overall mean PCV of positive *Trypanosoma evansi* camels was 22.2%, which was lower than the overall mean PCV of negative camels, which was 26.7% (Table 5).

The mean PCV for serologically and parasitologically positive and serologically and parasitologically negative camels was 22.1%, 21.7%, 26.6%, and 26.2%, respectively. Statistically significant (*p* ≤ 0.001) differences were recorded in parasitologically positive and negative camels (Table 6).

Out of 77 animals with positive serological test results, 25 were positive and 52 were negative using the Buffy coat method, respectively. Among the 35 animals with positive parasitological results, 25 were positive and 10 were negative using CATT/*T. evansi*, respectively, as shown in Table 7. A statistical test using kappa indicated a fair agreement between the two tests (*k* = 0.385). The relative sensitivity of the CATT/*T. evansi* test used in this study was found to be 25/77 (32.5%) (Table 7).

The prevalence of Trypanosoma evansi in camels of different ages, sex, body condition score, herd composition, season, and herd size is shown in Table 8. Among the risk factors considered, age, body condition score, and season appeared to have a significant effect (*p* ≤ 0.001) on the prevalence of *Trypanosoma evansi* in camels (Table 8).

Out of the camels infected with *Trypanosoma evansi*, 36.3% had *Tabanus* infestation, 33.3% had *Hippobosca* infestation, and 30.4% had *Stomoxys* infestation. The files were directly collected (caught) from the camels while the flies were feeding or resting on them by using aerial sweeping nets. *T. evansi*-infected camels were infested by at least one or more genera of biting flies. This indicates that the camels from which the flies were directly collected and blood samples tested highly positive for *T. evansi*. This finding demonstrated that some camels were concurrently affected by *Hippobosca*, *Stomoxys*, and *Tabanus*. This means that some camels have only one fly infested them, some have two flies infested them, and some have three flies infested them at the same time. *T. evansi* infection may be more likely to infect camels that biting flies directly caught by using aerial sweeping nets while they are feeding on the animals. The prevalence of the 3 genera was statistically significant (*p* < 0.05) (Table 9).

## 4. Discussion

The current study involving serological and parasitological examinations provided strong evidence that *Trypanosoma evansi* is highly prevalent and causes major constraints to the productivity and health of camels in the Amibara district of Gabi Rasu zone, Afar Regional State. The overall parasitological prevalence of 7.3% recorded in this study is in agreement with the previous findings in different parts of Ethiopia: (7.5%) [42], (6.5%), [43], (7.7%) [44], and 5% [45]. However, the findings of the present study are lower than previous reports of 12.5% [38], 12.1% [44], 10.2% [46], 10.9% [47], and 23.9% [48]. This difference might be linked to seasonal variation and agroecological conditions of the study areas.

The prevalence of trypanosomosis is determined by the seasonal and environmental factors, which are determined by the presence and number of biting flies. The occurrence of this condition is notably enhanced by the wet and humid weather conditions, as well as the increased presence of biting flies during the short rain and wet season when the population of these flies is abundant [11, 49]. *Tabanus* studies conducted in different tropical regions have shown a clear association between periodic occurrences of *T. evansi* infections. The study reveals variations in the distribution of camel biting flies among different flies' genera, consistent with previous research by [50].

During the rainy season, there is a rise in infections and an uptick in the fly population. Precipitation appropriate clay soil that retains moisture, as well as surface water pools, thus provides the necessary sustenance creating favorable environments for camels to graze, characterized by the abundant growth of *Acacia senegal* shrubs [11, 51].

A large proportion of camels were found to be seropositive, in addition to the parasitological findings. The current serological result is consistent with previous findings in different parts of Ethiopia (17.8%) in a similar study site by Weldegebrial et al. [42]. However, the current finding is lower than that of other researchers (24.9%) [44], 30% in Chad [52], 22% in India [53], 28% in Kenya [54], and 27.5% [55]. The present serological study result is higher than the previous study report conducted by the authors in [56] in Iran, which was 10%.

*Trypanosome* morphological identification in the current study showed that the amount of parasitemia varied from a few parasites per slide to over five t*rypanosomes* per field of view of the microscope. These findings coincide with the finding of the authors in [50]. The overall parasitological prevalence of *Trypanosoma evansi* in the current study area was 7.3%. This result was highly in agreement with the findings in [50], which reported 7.2% prevalence by using thin blood smears. The higher seroprevalence compared to the parasitological result recorded in the current study could be due to the fact that demonstration of *trypanosomes* in blood is quite unreliable because a large proportion of infections in the field (50 to 80%) do not develop detectable levels of parasitemia [57]. This is due to the fact that *trypanosome* infection in camels is usually chronic, with very low parasitemia. Additionally, the inability of the CATT/*T. evansi* test to distinguish current from cured infection [58], as detectable levels of antibodies can still be found in self-cured animals or after treatment with trypanocidal drugs [59], might contribute to the higher prevalence difference between the two tests.

CATT was sensitive in detecting 86 latent/aparasitaemic infections, but it failed to detect 10 of 35 (28.6%) patent/parasiteaemic infections (Table 7). The CATT/*T. evansi* test, a direct agglutination test, is the most widely used and has a proven record of reliability for various host species, including buffaloes and camels [60, 61]. This lower sensitivity of the CATT test observed in the current observation is consistent with previous studies in Kenya that reported a sensitivity of 65.5% [62] and 68.6% [15]. Similarly, Hagos et al. [44] reported a 72% sensitivity of CATT/*T. evansi* from Ethiopia. The occurrence of camel *trypanosomes* is more common in camels that are kept with other animals, such as cattle, sheep, and goats. This is similar to the observations made by the authors in [50], that camels herded with small ruminant animals like sheep and goats were found positive for *trypanosomes* through DNA identification using molecular techniques like PCR. The diverse range of animals that the biting flies feed on contributes to the observed epidemiological complexity of trypanosomosis [50].

The overall mean PCV of positive *Trypanosoma evansi* camels was 22.2%, which was lower than the overall mean PCV of negative camels, which was 26.7%. The current study showed that parasitologically negative camels had significantly higher mean PCV than positive camels, which is consistent with previous reports [44, 50, 63]. This suggests that the primary clinical finding of trypanosomosis's was anemia. In serological tests, there was no significant difference in mean PCV between serologically negative and serologically positive camels. This could be due to the CATT test's lack of ability to distinguish antibodies from active infections from those from cleared or past infections, as previously suggested by [58]. Therefore, the PCV values of cured camels from trypanosomosis (past infections) that are serologically detected as positive are not significantly different from seronegative ones [64], and highly reduced PCV values occur when trypanosome parasites are detectable in blood.

The higher prevalence of *T. evansi* in adults than in young camels in both parasitological and serological tests in the present study is in agreement with previous reports in [16, 60, 63, 65]. This could be because of larger-scale movement, which increases the risk of infection in adult camels [52, 66], heavy stress on adult male camels used for transportation of goods and possible poor management [67], and stress associated with pregnancy and lactation in adult female camels [66].

The present study demonstrated that biting flies are widespread and the most significant ectoparasites of camels in the Amibara district, Gabi Rasu zone, Afar region, Ethiopia. The observation of an overall prevalence of 87% biting flies in the present study is in line with the previous report of a 99.9% Stomoxys infestation in camels in and around the Fentale district [13]. This study also showed that the prevalence of Trypanosoma evansi has a correlation with seasonal increments in the number of Tabanus during the rainy season, which is in agreement with the previous reports [51, 68]. Experiments have shown that over 20 different *Tabanus* species can transmit *Trypanosoma evansi* [49]. The presence of *Tabanus* species across all study seasons suggests that parasite transmission occurs wherever reservoir hosts and susceptible host's coexist. The abundance of *Tabanus* species has been linked to sporadic outbreaks of the disease during the dry season and outbreaks during the rainy season [51]. Some investigators state that Stomoxys and Hippobosca are possible vectors for transmission of Trypanosoma evansi [47, 69].

Although *Stomoxys* has been implicated as a vector of *Trypanosoma evansi*, experimental trials involving transmission between horses, guinea pigs, and dogs have not proved that these flies are important vectors [36, 70]. However, it has been reported that the efficiency of different flies in transmitting *Trypanosoma evansi* varies depending on geographic conditions, as well as the interval between two successive feeds and the intensity of the fly challenge [49]. Experimental trials conducted on *Hippobosca camelina* in Kenya to assess the mechanical transmission of *Trypanosoma evansi* showed that the fly failed to transmit the pathogen, but regurgitation is suspected to be the other possible means of transmission [29]. However, *Hippobosca camelina* and *Stomoxys calcitran* are the primary carriers of trypanosomes in regions devoid of tsetse flies, according to research in the report of [50]. This is because *trypanosomes* are consistently found in them, and they are abundant throughout the year. Additionally, in the current study, there is a notable preference for *Hippobosca* in camels, which corresponds to the finding reported by [50]. The study mentioned above revealed *that Hippobosca camelina* had a clear inclination towards camels, as most of them were found to feed on camels. According to Getahun et al. [50], it is confirmed that *Hippobosca camelina*, *Stomoxys calcitrans*, and *Tabanus* spp were identified as the potential vectors of *trypanosomes* outside of the tsetse fly belt.

In the present study, among the camels that were found to be infected with *Trypanosoma evansi*, 36.3% were infested with *Tabanus*, 33.3% were infested with *Hippobosca*, and 30.4% were infested with *Stomoxys*. During the study period, the files were captured directly using aerial sweeping nets, and blood samples were simultaneously extracted from them. Camels infected with *T. evansi* were infested by one or more types of biting flies at the same time. This suggests that the camels, from which the flies were directly collected and blood samples were tested, had a high rate of positivity for *T. evansi*. Based on the current study findings, *T*. *evansi* infection is more likely to occur in camels when they are bitten by flies that are directly caught using aerial sweeping nets. The remaining flies were also collected by using aerial sweeping nets while the flies were flying or circling around the animals in the study area. In the result section of Table 9, we focus only on the biting flies collected by aerial sweeping nets directly from the camels. This is due to the fact that the research paper primarily focuses on biting flies and their associated pathogens in the study area.

The finding of a higher prevalence of biting flies as well as *Trypanosoma evansi* in adult camels than in the young camels in the present study might be most probably attributed to the fact that pastoralists kept young camels in the residential area and they do not go to distant areas where the fly burden is high.

## 5. Conclusion and Recommendations

Hippobosca and Stomoxys are the most prevalent biting flies in the study area. Trypanosoma evansi is one of the most common pathogens affecting camels in the study area. Therefore, it is necessary to make significant endeavors at various levels to minimize the adverse effects on the health, production, and output of camels in the district. This is crucial for enhancing the living standards of the pastoralist community residing in the research area, where camels are predominantly used for milk, meat, and as a source of income. Therefore, based on the above conclusion, the following recommendations are forwarded:

- Practically applicable control interventions need to be implemented to reduce the negative impacts of biting flies and trypanosomosis in camels with the appropriate use of acaricides in the study area
- Advanced molecular studies on biting flies' identification to species level and their associated pathogens in animal diseases in the wider pastoralist community in various parts of Ethiopia are needed

## Figures and Tables

**Table 1:** Relative abundance of camel biting flies at the genus level in the Amibara district.

Genus of biting flies	Frequency (*N*)	Percent (%)
*Hippobosca*	689	40.4
*Stomoxys*	529	31.00
*Tabanus*	448	28.6
Total	1,706	100

**Table 2:** Overall abundances of collected biting flies in camels throughout different seasons in the Amibara district.

Season	Genus of biting flies			
	*Hippobosca*	*Stomoxys*	*Tabanus*	Total
Wet	234	159	143	536
Dry	195	142	110	447
Short rainy	260	228	235	723
Total	689	529	488	1,706

**Table 3:** Prevalence of biting flies on camels in the Amibara district by different risk factors.

Variable	Category	No. of examined	No. of positive (%)	*x* ^2^	*p* value
Sex	Male	115	97 (84.3)	1.0061	0.316
	Female	365	321 (88)		

Age	Young	200	167 (83.5)	3.9138	0.048^∗^
	Adult	280	251 (89.6)		

Body condition	Poor	226	197 (87.2)	0.0504	0.975
	Good	111	96 (86.5)		
	Medium	143	125 (87.4)		

Herd composition	Mixed	234	202 (86.3)	0.2336	0.629
	Kept separately	246	216 (87.8)		

Season	Short rain	160	148 (92.5)	8.0383	0.018^∗^
	Wet	160	139 (86.8)		
	Dry	160	131 (8.7)		

Herd size	<20	87	75 (81.8)	0.2528	0.881
	20–40	162	140 (86.4)		
	>40	231	203 (88)		

^*∗*^ = reference category, *x*^*2*^ = chi square.

**Table 4:** Overall parasitological and serological prevalence of *Trypanosoma evansi* in camels in the Amibara district in Afar Region, Ethiopia.

No. of examined	Parasitological prevalence (no. of positive)	Serological prevalence (no. of positive)
480	7.3% (35)	16% (77)

**Table 5:** Overall mean PCV of parasitologically positive and negative camels in the Amibara district Afar Region, Ethiopia.

Mean PCV	
Parasitologically positive (%)	Parasitologically negative (%)
22.2	26.7

**Table 6:** Mean PCV of camels positive and negative for parasitological and serological trypanosomosis in the Amibara district, Afar Region, Ethiopia.

Camel trypanosomosis status	No. of observations	Mean PCV	Std. error	*p* value
CATT−	403	26.6	0.09	0.074
CATT+	77	22.1	0.21	
BCM−	445	26.2	0.27	
BCM+	35	21.7	0.36	≤0.001

CATT−: serological negative; CATT+: serological positive; BCM−: parasitological negative; BCM+: parasitological positive.

**Table 7:** Comparative positivity for *Trypanosoma evansi* in camels using parasitological and serological type of tests in the Amibara district, Gabi Rasu zone, Afar Region, Ethiopia.

Tests		Parasitological (BCM)				
		Positive	Negative	Total	Kappa value	*p* value
Serological	Positive	25	52	77	0.385	≤0.001
	Negative	10	393	403		
	Total	35	445	480		

BCM = Buff coat method.

**Table 8:** Overall prevalence of *Trypanosoma evansi* in camels with respect to different risk factors in the Amibara district.

Variable	Category	No. of examined	No. of positive (%)	*x* ^2^	*p* value
Sex	Male	115	16 (14)	1.4949	0.221
	Female	365	71 (19.4)		

Age	Young	200	17 (8.5)	22.1753	≤0.001^∗^
	Adult	280	70 (25)		

Body condition	Poor	226	63 (27.8)	31.7405	≤0.001^∗^
	Good	111	4 (3.6)		
	Medium	143	20 (14)		

Herd composition	Mixed	234	48 (20.5)	1.1911	0.275
	Kept separately	246	39 (15.8)		

Season	Short rain	160	56 (35)	36.2270	≤0.001^∗^
	Wet	160	17 (10.6)		
	Dry	160	14 (8.7)		

Herd size	<20	87	11 (12.6)	0.1957	0.907
	20–40	162	30 (18.5)		
	>40	231	46 (20)		

^*∗*^ = reference category, *x*^2^ = chi square.

**Table 9:** Camels were positive for biting flies and *Trypanosome evansi* simultaneously in the Amibara district.

Genus	No. of camels infested by flies and positive for *T. evansi*	Percent (%)	*x* ^2^	*p* value
*Tabanus*	87	36.3	80.4170	≤0.001
*Hippobosca*	80	33.3	8.6897	0.003
*Stomoxys*	73	30.4	1.387	0.004

## Data Availability

The data used to support the findings of this study are available from the corresponding author upon request.
